# Supplementary material for: Improvement of the Clinical and Psychological Profile of Patients with Autism after Methylcobalamin Syrup Administration
Source: Nutrients. 2022 May 12;14(10):2035. doi: 10.3390/nu14102035 (PMC9144375; doi:10.3390/nu14102035)
Supplement: Supplementary file 1 [file nutrients-14-02035-s001.zip › Supplementary File S1A.pdf]

Supplement 1A

Comparison of total scores of clinical and psychological profile provided by Psychologist and By Parents (Bivariate Fit and Passing Bablok regression)

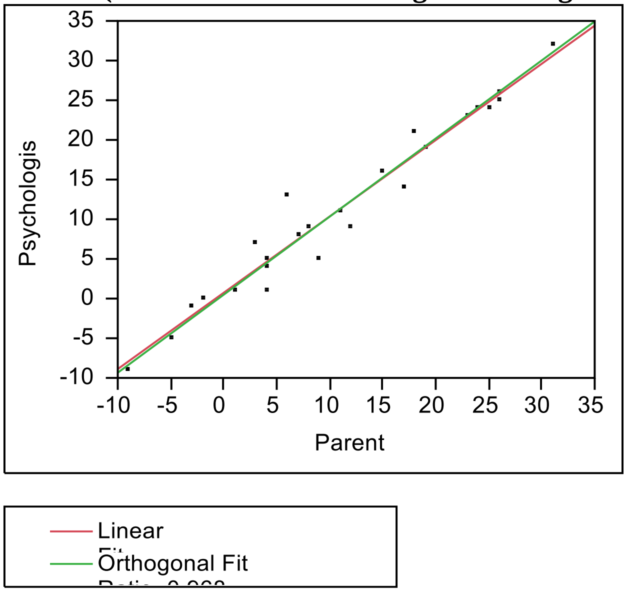

Linear Fit

Psychologist = 0,7367192 + 0,9619782*Parents

Summary of Fit

RSquare	0,955576
RSquare Adj	0,953645
Root Mean Square Error	2,333422
Mean of Response	11,28
Observations (or Sum Wgts)	25

Analysis of Variance

Source	DF	Sum of Squares	Mean Square	F Ratio
Model	1	2693,8083	2693,81	494,7435
Error	23	125,2317	5,44	**Prob > F**
C. Total	24	2819,0400		<,0001*

Parameter Estimates

Term	Estimate	Std Error	t Ratio	Prob>|t|
Intercept	0,7367192	0,66519	1,11	0,2795
Parents	0,9619782	0,043249	22,24	<,0001*

Orthogonal Regression

Variable	Mean	Std Dev	Variance Ratio	Correlation
Parents	10,96	11,01317	0,968423	0,9775
Psychologist	11,28	10,8379		

Intercept	Slope	LowerCL	UpperCL	Alpha
0,494431	0,984085	0,896447	1,08029	0,05000

Comparison of total scores of psychological and clinical evaluation provided by Psychologist and By Parents (Matched Pairs on Difference: Psychologist-Parents)

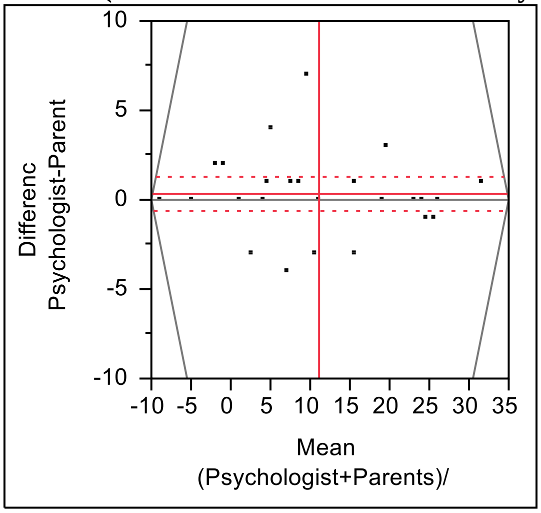

Psychologist	11,28	t-Ratio	0,688956
Parents	10,96	DF	24
Mean Difference	0,32	Prob > |t|	0,4975
Std Error	0,46447	Prob > t	0,2487
Upper 95%	1,27862	Prob < t	0,7513
Lower 95%	-0,6386		
N	25		
Correlation	0,97754		

Comparison of total scores of psychological and clinical evaluation provided by Psychologist and By Parents (Distributions of Difference Psychologist - Parents)

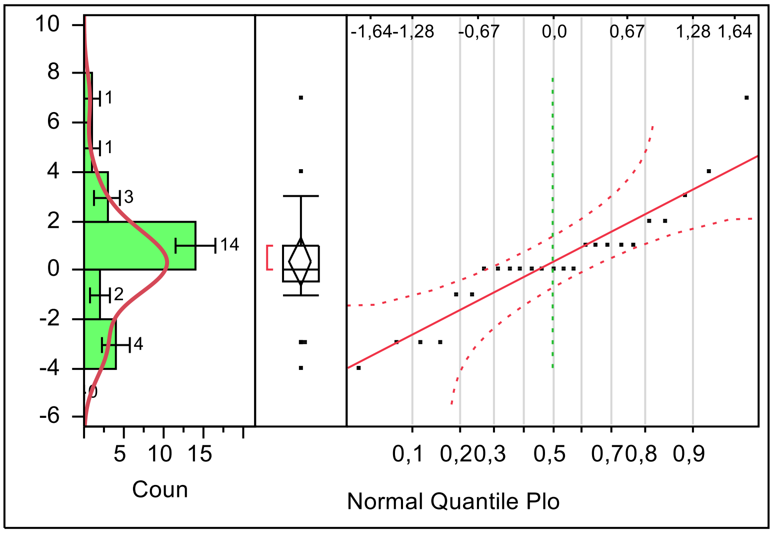

Smooth Curve

Nonparametric Density

Kernel Std

1,097951

Summary Statistics

Mean	0,32
Std Dev	2,3223551
Std Err Mean	0,464471
Upper 95% Mean	1,2786211
Lower 95% Mean	-0,638621
N	25
CV	725,73598
Median	0
Mode	0

Bivariate Fit of Percentile (Psychologist - Parents) By Difference (Psychologist - Parents)

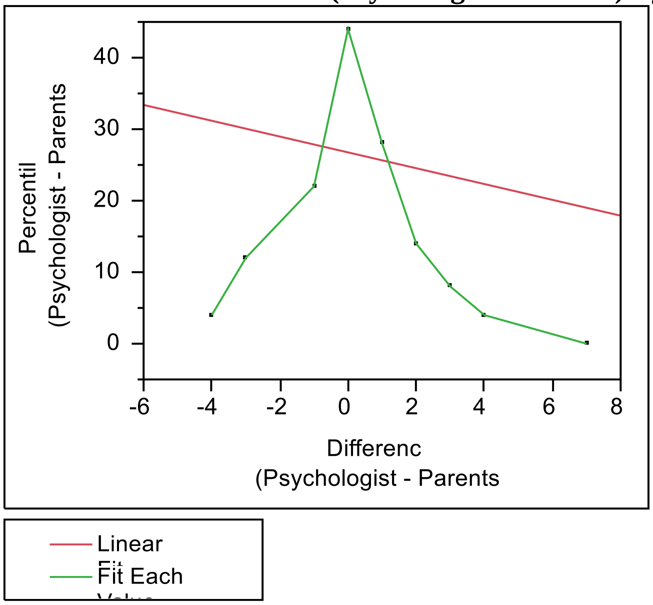

hhhhhhh

Linear Fit

Percentile (Psychologist - Parents) = 26,754017 - 1,1063041*Difference (Psychologist - Parents)

Summary of Fit

RSquare	0,027277
RSquare Adj	-0,01502
Root Mean Square Error	15,67271
Mean of Response	26,4
Observations (or Sum Wgts)	25

Analysis of Variance

**Source**	**DF**	**Sum of Squares**	**Mean Square**	**F Ratio**
Model	1	158,4227	158,423	0,6450
Error	23	5649,5773	245,634	**Prob > F**
C. Total	24	5808,0000		0,4301

Parameter Estimates

**Term**	**Estimate**	**Std Error**	**t Ratio**	**Prob>|t|**
Intercept	26,754017	3,165387	8,45	<,0001*
Difference (Psychologist - Parents)	-1,106304	1,377558	-0,80	0,4301
