# Supplementary material for: Improvement of the Clinical and Psychological Profile of Patients with Autism after Methylcobalamin Syrup Administration
Source: Nutrients. 2022 May 12;14(10):2035. doi: 10.3390/nu14102035 (PMC9144375; doi:10.3390/nu14102035)
Supplement: Supplementary file 1 [file nutrients-14-02035-s001.zip › Supplementary File S1B.pdf]

Supplement 1B. Variability Chart for Overall change at d200

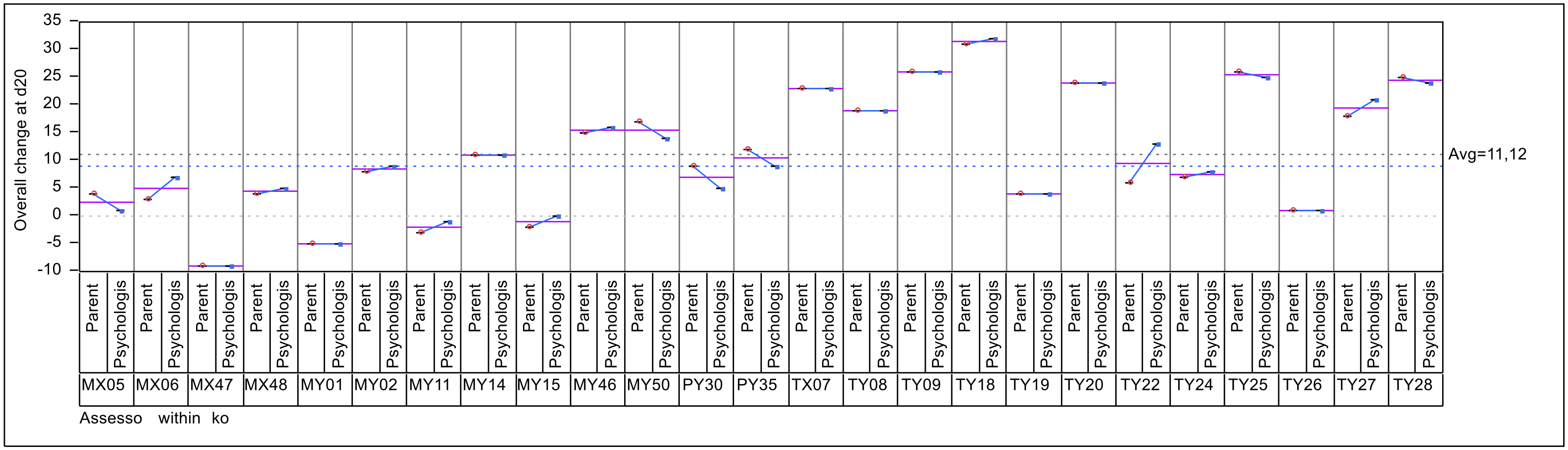

Switching to Bayesian estimates because of negative REML variance component(s).

Bayesian Variance Component Estimates

Random Effect	Var Component	Pct of Total
kod	107,39685	97,751
Assessor	0,0696497	0,063
kod*Assessor	2,4013848	2,186
Total	109,86788	100,000

Residual is confounded with kod*Assessor and has been removed.

Variance Components

Component	Var Component	% of Total	Plot%	Sqrt(Var Comp)
kod	107,39685	97,8		10,363
Assessor	0,06965	0,0634		0,264
kod*Assessor	2,40138	2,2		1,550
Within	0,00000	0,0		0,000
Total	109,86788	100,0		10,482
