# Supplementary material for: Improvement of the Clinical and Psychological Profile of Patients with Autism after Methylcobalamin Syrup Administration
Source: Nutrients. 2022 May 12;14(10):2035. doi: 10.3390/nu14102035 (PMC9144375; doi:10.3390/nu14102035)
Supplement: Supplementary file 1 [file nutrients-14-02035-s001.zip › Supplementary File S2.pdf]

Supplement 2. Scatter plot of GSH levels

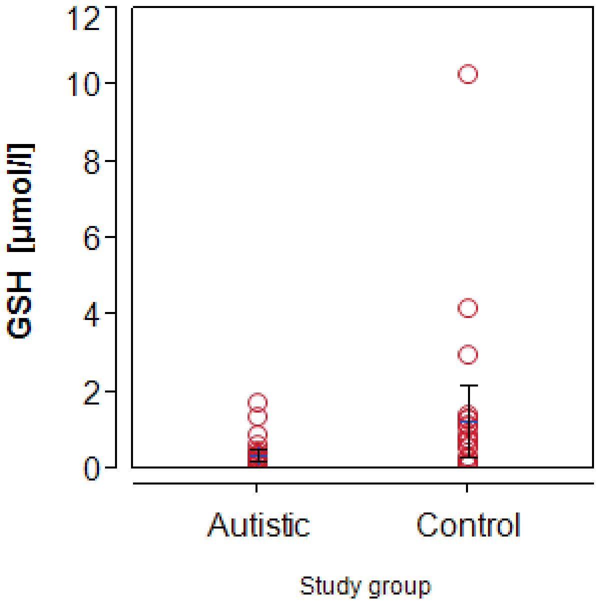

Comparison of the level of GSH in autistic patients (0.36 ± 0.40 µmol/l, n=25) and in the control group (1.24 ± 2.20 µmol/l, n=23)
