# Supplementary material for: Improvement of the Clinical and Psychological Profile of Patients with Autism after Methylcobalamin Syrup Administration
Source: Nutrients. 2022 May 12;14(10):2035. doi: 10.3390/nu14102035 (PMC9144375; doi:10.3390/nu14102035)
Supplement: Supplementary file 1 [file nutrients-14-02035-s001.zip › Table S1.pdf]

Supplement 1. An assessment scale with 54 items to address the main social (13 items), communication (10 items), behavioral (14 items), and cognitive (17 items) features that are known to be affected in patients with autism.

**SOCIAL CATEGORIES**<br>The items observe and evaluate typical clinical signs of social disturbance present in autism and the manifestations of social and communication behaviors of an autistic individual. The categories were taken from classification criteria (ICD-10, DSM-IV) and accepted diagnostic methods like ADI-R [1,2], CARS-2 [3], Autistic Continuum [4], and the clinical praxis.	
**tolerance of physical contacts**<br>- we evaluate whether the child tolerates, or refuses physical contact; we do not evaluate whether he or she reciprocates the contact	
**interest in physical contact**<br>- we evaluate whether the child actively seeks out physical contact; elements of social reciprocity must be present; physical routines are not included in this category	
**tolerance of peers**<br>- we evaluate whether the child tolerates peer group, and whether he or she is able to participate in parallel activities (i.e., the child is able to play near other children without signs of aggression and destructivity)	
**active interest in peers**<br>- we evaluate whether the child participates and/or cooperates in peer activities (this category does not include routine motor behaviors -- e.g., playing catch-up, swinging, sliding, etc., as these may represent isolated activities)	
**social aggression**<br>- we evaluate whether the child uses signs of social aggression like kicking, hitting, pinching to initiate contact with another person	
**social destructivity**<br>- we evaluate whether the child uses signs of social destructivity like destroying or throwing objects, banging doors in social situations, refusal to enter new environment due to being overloaded by social stimuli	
**solitary activities**<br>- we evaluate social qualities of the child's play and other activities, whether he or she prefers solitary play; we also evaluate whether the child actively seeks out opportunities to engage in solitary activities, e.g., by leaving social situation	
**passivity, lack of interest**<br>- we evaluate whether the child is isolated, passive, and needs pressure to start or change an activity	
**sensitivity to social environment**<br>- we evaluate how the child reacts to the presence of several people in his or her natural environment (e.g., we observe the child's behavior in social situations like public events, shopping, traveling, etc.)	
**cooperative play**<br>- we evaluate whether the child is interested in mutual activities (peer games, cooperation with another person, e.g., play with a parent or a therapist)	
**motivation to verbalize**<br>- we evaluate whether the child tries to use his or her verbal abilities (word approximations, sounds, interjections, etc.) in contact with another person (this item evaluates the use of "verbalizations" as a primary means of communication)	
**feedback on information from another person**	

- we evaluate whether the child reacts to information (verbal information, activities initiated by another person) and is able to modify his or her activity accordingly	
**respecting communication partner**<br>- we evaluate whether the child is able to use his or her verbal abilities to participate in simple conversation (if the child is non-verbal, than evaluate alternative forms of communication, e.g., gestures, pictograms, symbols)	
**COMMUNICATION CATEGORIES**<br>The items observe and evaluate typical clinical signs of communication disturbance present in autism and the manifestations of social and communication behavior of an autistic individual. The categories were taken from classification criteria (ICD-10, DSM-IV) and accepted diagnostic methods like ADI-R [2], CARS-2 [3], Autistic Continuum [4], and the clinical praxis.	
**reaction to emotional behavior of another person**<br>- we evaluate whether the child is sensitive and reacts to loud laughter or speech of another person or group of people (we evaluate all reactions, e.g., covering his or her ears, interrupting the emotions of other people by loud cry, fascination by the emotion, etc.)	
**pathological forms of attention**<br>- we evaluate the presence of behaviors intended to attract attention of another person (mild destructivity, cry, throwing oneself to the ground, throwing objects, aggression towards another person, etc.)	
**the use of gestures**<br>- we evaluate whether the child uses basic communication modalities like eye contact, pointing, subjective gestures (eat, drink, etc.), and the use of objects is also accepted, e.g.,the child uses objects to share information	
**the use of behavior to express need or interest (pre-communication)**<br>- we evaluate whether the child uses atypical behaviors to indicate interest or need (may be negative), that are known, e.g., a parent understands them, but another person does not. Temper tantrums (e.g., crying, if favorite activity is terminated), destructivity, aggression (as a reaction to the child's failure, e.g., failure to understand a piece of information), throwing oneself to the ground, the use of another person's hand or body part are also evaluated here	
**the presence of words, sentences, verbalizations**<br>- we evaluate whether the child uses verbalizations, all levels of verbalizations are evaluated (e.g., playing with sounds, mechanical memorization of stories, replaying texts, etc.)	
**the presence of delayed echolalia**<br>- we evaluate the presence of delayed echolalia in verbal utterances of the child (non-functional reproductions of texts, memorizing of poems or fairy tales, etc.)	
**the presence of immediate echolalia**<br>- we evaluate the presence of immediate echolalia (the child mechanically repeats a word or a sentence in direct contact with another person)	
**the level of understanding of simple information**<br>- we evaluate whether the child understands simple information in a real-life context ("Do you want a drink?", "Get dressed.", "Give me the toy car." "We are going out."...)	

**the level of understanding of complex information**<br>- we evaluate whether the child understands more complex information (the explanation of a change in activity, demanding a change of behavior in changing context, basic conditioning, e.g., "if you wait, you will get a toy")	
**appropriate use of verbalization**<br>- we evaluate how well the child is able to use his or her verbal abilities (even if the child uses only one-word sentences)	
BEHAVIORAL CATEGORIES<br>The items observe and evaluate typical autistic behaviors as a result of primary deficits in childhood autism (typical triad of impairments, ICD-10). The categories were taken from classification criteria (ICD-10, DSM-IV) and accepted diagnostic methods like ADI-R [2], CARS-2 [3], Autistic Continuum [4], and the clinical praxis.	
**"motor emotions"**<br>- we evaluate the presence of motor stereotypies (hand flapping, body rocking, jumping, etc.), which are connected with the emotions of the child (e.g., during sensory activities, favorite stereotyped behaviors, etc.)	
**the presence of routines**<br>- we evaluate whether the child demands the same routines or activities (daily regime, putting objects at the same place, preferring one sensory quality of an object) in selected contexts (e.g., at home, at school)	
**the presence of rituals**<br>we evaluate whether the child demands more complex sequences of activities (e.g., leaving home, traveling, the routes of walks, sequences of actions while dressing, eating, etc.) from another person in selected contexts	
**lack of flexibility**<br>- we evaluate whether the child can divert attention from routine or ritual	
**stereotyped behaviors**<br>- we evaluate the presence of typical autistic qualities in the child's play, e.g., the presence of manipulations (lining up, sorting, rotating, sifting, etc.)	
**aggression**<br>- we evaluate the presence of aggressive behaviors (pinching, kicking, biting, hitting, etc.)	
**destructivity**<br>- we evaluate the presence of destructive behaviors (destroying objects, throwing objects, deliberate dismantling of objects - "studying them", etc.)	
**self-injurious behaviors**<br>- we evaluate the presence of self-injurious behaviors (head banging, hand biting, eye pressing, hair pulling, etc.)	
**tantrums**<br>- we evaluate the presence of unusually intense temper tantrums, with or without a cause	
**hyperactivity**<br>- we evaluate the presence of hyperactivity, e.g., running, climbing, escaping	
**motor stereotypies**<br>- we evaluate the presence of typical autistic motor stereotyped behaviors like toe walking, spinning around one's axis, circling around objects, running up and down, rocking, etc.	

**psychomotor sensitivity**<br>- we evaluate whether the child is sensitive to psychomotor stimuli, meaning whether there occurs a significant change after socio-communication and sensory load (e.g., spending time in broader social context, the disruption of daily routines of the child, intensive stimulation, etc.) like the onset of hyperactivity, disorganization, temper tantrums, seeking solitude, and the absence of reactions, e.g., "shutdown"	
**the presence of physiological disorders**<br>- we evaluate the presence of sleep disorders (insomnia, short sleep, discontinuous sleep) or eating disorders (referring not to hypersensitivity, but to the amount of food)	
**independence in simple activities**<br>- we evaluate whether the child is able to perform simple tasks (age-appropriate - eating, drinking, hygiene - without the assistance of another person or with only a little assistance)	
**COGNITIVE QUALITIES**<br>The items observe and evaluate typical clinical signs of cognitive disturbance present in autism (specific qualities of perception, disturbance of imagination, hypotrophy and hypertrophy of cognitive abilities) with influences on behavior (especially the sphere of performance, mastering new abilities and using them in various contexts) and the level of functioning in the natural environment. The categories were taken from classification criteria (ICD-10, DSM-IV) and accepted diagnostic methods like ADI-R [2], CARS-2 [3], renowned neuropsychological studies [5–8] and clinical praxis.	
**simple symbolic behaviors (imagination, imitation)**<br>- we evaluate whether the child tries to imitate simple symbolic behaviors, e.g., adequately uses toy car; we accept play cooking or using a phone; we evaluate especially imitation of the activities from the environment	
**fascination by stimuli/objects**<br>- we evaluate whether the child prefers objects and stimuli (sensory) from the environment to contact with people and their information (non-social category)	
**shifting attention from stimuli, activity (distribution of attention)**<br>- we evaluate whether the child is able to occupy himself or herself with one stimulus or activity; whether he or she is unable to shift his or her attention to another stimulus or is able to shift his or her attention only with severe difficulties	
**short-time interest in stimuli (the capacity of attention)**<br>- we evaluate the presence of problems with holding attention for some time; we evaluate whether the child is able to occupy oneself with the object as expected	
**reaction to stimuli from another party (vigilance of attention, non-social category)**<br>- we evaluate the vigilance of attention with a focus on the child's reactions to stimuli from another person, e.g., reaction to information from another party, interest in stimuli being offered, shifting of attention, motivation to something other than subjectively preferred activities (optimally evaluate during work, therapy or education)	
**attention to detail (fragmentation)**<br>- we evaluate the level of fragmentation of attention, whether the child deliberately seeks out parts of objects (e.g., wheels, lights, buttons, selected motifs on pictures, parts of objects, etc.)	
**good/bad performance (executive functioning, short-time memory)**<br>- we evaluate whether the child is able to perform meaningful activities (e.g., developmental tasks during the process of education, basic daily activities like getting	

dressed, hygiene, basic tasks like housework, etc.) independently in the right sequence so the activity is being performed without significant support from another person	
**sensitivity to sounds (hypersensitivity)**<br>- we evaluate whether the child is sensitive to sound stimuli from the environment	
**sensitivity to various sensory stimuli**<br>- we evaluate the presence of hypersensitivity to various sensory stimuli, e.g., fascination by lights, smells, materials that influence activities, interests and actions of the child (and are frequently the core of the child's play)	
**picky eating (hypersensitivity)**<br>- we evaluate whether the child is too picky; whether he or she tolerates only selected food	
**sensitivity to clothing and other objects (hypersensitivity)**<br>- we evaluate whether the child refuses selected materials and types of clothing	
**sensory self-stimulation (qualities of perception)**<br>- fascination by sensory stimuli, child may "isolate himself or herself" (when the child is occupied by the object, he or she fails to react-- "shuts down"), he or she chooses activities that fulfill his or her need (looking at objects from unusual angles, looking out of the window, watching the trees moving in the wind, etc.)	
**sensory discrimination (visual hyper-decoding)**<br>- we evaluate the presence of excellent discrimination abilities of the child (especially visual) - fascination by logos, marks, letters, numbers (which are not used functionally); sorting by colors or shapes	
**symbolic discrimination (imagination)**<br>- we evaluate the level of understanding and discrimination of concepts (sorting objects according to common characteristics - meaning, context)	
**academic abilities (item depends on the child's chronological age)**<br>- the presence, absence or onset of academic abilities in time (knowing letters, numbers, mathematic and lexical abilities)	
**the use of knowledge (generalization)**<br>- the child is able to use his or her abilities in various contexts (e.g., he or she is able to get dressed at school as well as at home, he or she is able to wait at school as well as in another environment, he or she is able to use academic knowledge (e.g.,count objects, read simple information and perform an activity, etc.)	
**imitation**<br>- the child is able to imitate simple activities in real-life settings (home, school), this item is evaluated according to the child's chronological age and developmental level	

References
1. Lord C, Rutter M, Le Couteur A. Autism Diagnostic Interview-Revised: a revised version of a diagnostic interview for caregivers of individuals with possible pervasive developmental disorders. *J Autism Dev Disord*. 1994;24(5):659-685.

2. Rutter M, LeCouteur A, Lord C. *(ADI^{TM}-R) Autism Diagnostic Interview^{TM}, Revised*. 4. the United States of America: Western Psychological Services; 2010.

3. Schopler E, Van Bourgondien ME, Wellman J, Love SR. *CARS-2. Childhood Autism Rating Scale.* 4. the United States of America: Western Psychological Services; 2011.

4. Wing L. *Autistic Continuum Disorders*. 2nd ed. London: The National Autistic Society; 1993.

5. Bailey A, Phillips W, Rutter M. Autism: towards an integration of clinical, genetic, neuropsychological, and neurobiological perspectives. *J Child Psychol Psychiatry*. 1996;37(1):89-126.

6. Filipek PA, Accardo PJ, Ashwal S, et al. Practice parameter: screening and diagnosis of autism: report of the Quality Standards Subcommittee of the American Academy of Neurology and the Child Neurology Society. *Neurology*. 2000;55(4):468-479.

7. Happé F, Frith U. The neuropsychology of autism. *Brain J Neurol*. 1996;119 (Pt 4):1377-1400.

8. Ozonoff S, Goodlin-Jones BL, Solomon M. Evidence-based assessment of autism spectrum disorders in children and adolescents. *J Clin Child Adolesc Psychol Off J Soc Clin Child Adolesc Psychol Am Psychol Assoc Div 53*. 2005;34(3):523-540. doi:10.1207/s15374424jccp3403_8
